# Willingness to pay for kidney transplantation among chronic kidney disease patients in Ghana

**DOI:** 10.1371/journal.pone.0244437

**Published:** 2020-12-30

**Authors:** V. Boima, K. Agyabeng, V. Ganu, D. Dey, E. Yorke, M. B. Amissah-Arthur, A. A. Wilson, A. E. Yawson, C. C. Mate-Kole, J. Nonvignon

**Affiliations:** 1 Department of Medicine and Therapeutics University of Ghana Medical School, College of Health Sciences, University of Ghana, Accra, Ghana; 2 Departments of Biostatistics, School of Public Health, College of Health Sciences, University of Ghana, Accra, Ghana; 3 Department of Medicine and therapeutics, Korle-Bu Teaching Hospital, Accra, Ghana; 4 Departemnt of Public Health, Greater Accra regional Hospital, Ghana Health Service, Accra, Ghana; 5 Department of Psychology/Center for ageing studies, College of Humanities, University of Ghana, Legon, Ghana; 6 Department of Health Policy, Planning and Management, School of Public Health, College of Health Sciences, University of Ghana, Accra, Ghana; Imperial College Healthcare NHS Trust, UNITED KINGDOM

## Abstract

**Background:**

Kidney transplantation is the preferred treatment for patients with end stage renal disease. However, it is largely unavailable in many sub-Sahara African countries including Ghana. In Ghana, treatment for end stage renal disease including transplantation, is usually financed out-of-pocket. As efforts continue to be made to expand the kidney transplantation programme in Ghana, it remains unclear whether patients with Chronic Kidney Disease (CKD) would be willing to pay for a kidney transplant.

**Aim:**

The aim of the study was to assess CKD patients’ willingness to pay for kidney transplantation as a treatment option for end stage renal disease in Ghana.

**Methods:**

A facility based cross-sectional study conducted at the Renal Outpatient clinic and Dialysis Unit of Korle-Bu Teaching Hospital among 342 CKD patients 18 years and above including those receiving haemodialysis. A consecutive sampling approach was used to recruit patients. Structured questionnaires were administered to obtain information on demographic, socio-economic, knowledge about transplant, perception of transplantation and willingness to pay for transplant. In addition, the INSPIRIT questionnaire was used to assess patients’ level of religiosity and spirituality. Contingent valuation method (CVM) method was used to assess willingness to pay (WTP) for kidney transplantation. Logistic regression model was used to determine the significant predictors of WTP.

**Results:**

The average age of respondents was 50.2 ± 17.1 years with most (56.7% (194/342) being male. Overall, 90 out of the 342 study participants (26.3%, 95%CI: 21.7–31.3%) were willing to pay for a kidney transplant at the current going price (≥ $ 17,550) or more. The median amount participants were willing to pay below the current price was $986 (IQR: $197 –$1972). Among those willing to accept (67.3%, 230/342), 29.1% (67/230) were willing to pay for kidney transplant at the prevailing price. Wealth quintile, social support in terms of number of family friends one could talk to about personal issues and number of family members one can call on for help were the only factors identified to be significantly predictive of willingness to pay (p-value < 0.05).

**Conclusion:**

The overall willingness to pay for kidney transplant is low among chronic kidney disease patients attending Korle-Bu Teaching Hospital. Patients with higher socio-economic status and those with more family members one can call on for help were more likely to pay for kidney transplantation. The study’s findings give policy makers an understanding of CKD patients circumstances regarding affordability of the medical management of CKD including kidney transplantation. This can help develop pricing models to attain an ideal poise between a cost effective but sustainable kidney transplant programme and improve patient access to this ultimate treatment option.

## Introduction

Chronic kidney disease (CKD) is a major public health problem with global prevalence between 11.7% to 15.1% [1]. In 2015, the global burden of disease (GBD) study showed that 1.2 million people died from kidney disease representing 32% increase in mortality since 2005 [2]. In 2010, 2.3–7.1 million people with end stage renal disease (ESRD) died without access to dialysis [3]. As a result, the overall annual mortality due to kidney disease is approximately 5–10 million deaths. In addition, Disability Adjusted Life Years (DALYs) linked to kidney disease increased from 19 million in 1990 to 33 million in 2013 [4]. In view of limited epidemiological data, lack of awareness and poor access to laboratory services, the true burden posed by kidney disease is likely underestimated in Africa.

Estimates show that 78% of the 500 million people affected globally by CKD are in low- and middle-income countries (LMICs). The prevalence of CKD in these LMICs is 14.3% in the general population and 36.1% in high-risk populations [5,6]. The average prevalence in sub-Sahara Africa is 13.9% with prevalence between 2.0% in Côte d’Ivoire and 30% in Zimbabwe [7]. The estimated prevalence in Ghana is 13.3% [8].

The economic burden associated with kidney disease is high compared to other chronic diseases [9]. High-income countries usually spend more than 2–3% of their annual income on treatment of end stage renal disease [9]. Kidney transplant is the gold standard among the treatment options available to patients. It is the preferred option in LMICs where cost of treatment is a major obstacle [10]. In high countries the transplant rate is between 30–50 per million population (pmp) compared to LMICs where the transplant rate is between 0–10 pmp [11]. In Africa, the transplant rate averages between 4 and 7.2 pmp [10].

Although kidney transplantation is the preferred treatment, the predominantly available option for renal replacement therapy in Ghana is haemodialysis. Kidney transplantation which started as a pilot project in Ghana in 2008, is still waiting establishment of formal legislation to guide the development of a sustainable transplant programme [12]. Ghana currently performs only living related donor kidney transplant.

A recent study in Ghana indicated that 85.4% of study participants were willing to accept kidney transplantation [13]. All ESRD patients receive regular education on modalities of renal replacement therapy including dialysis and transplant at every clinic visit. Educational information provided include cost and type of treatment, risks and advantages of kidney transplantation. In addition, pre-transplant education emphasizes long term immunosuppressive therapy and its associated cost implications.

However, payment of healthcare cost in Ghana is either out-of-pocket or by the existing National Health Insurance Scheme (NHIS), which has coverage of about 40% and is social insurance scheme funded by tax, social security and premiums from mainly informal sector workers. Though the scheme covers a range of health services, it does not cover health services like organ transplantation, dialysis and cost of immunosuppressive drugs, [14]. As a result, the cost of dialysis and transplant are borne by respective individuals, organizations and philanthropists [12]. The estimated cost of kidney transplantation in Ghana was $18,000 in 2014 compared to the estimated cost of $14000 in 2008 [12]. At the time of conducting this study, the cost of kidney transplantation in Ghana was $17,550. Varying discounts to a maximum of 15% discount ($14918) were made available for low income and needy patients who require kidney transplantation. In addition, the current cost per session of dialysis in Ghana ranges from $60 - $72 and the cost of three dialysis sessions per week is US$149.10, representing a high financial burden on the patient and family. For this kidney transplant programme to be sustainable, there is a need to assess the willingness of CKD patients to pay for this service.

The aim of this study was to examine willingness of CKD patients in Ghana to pay for kidney transplantation. Further, it assess the factors associated with their WTP for this health service using Contingent Valuation Method (CVM), a validated tool used widely to access WTP for health services.

## Materials and methods

The present study, a facility-based cross-sectional study with a quantitative approach was conducted April and May 2019, among CKD patients attending the renal outpatient clinic and the haemodialysis units of the Korle-Bu Teaching Hospital (KBTH) in Accra. The KBTH is a national referral hospital that provides services for many patients including patients with CKD and ESRD patients receiving renal replacement therapy (haemodialysis and kidney transplant).

Using the Cochran's (1977) formula, the minimum required sample size for this study was estimated as n=Zα22×P×(1−P)d2 (n = minimum required sample size; α = Significance level = 5%; Zα22 = z-score at 95% confidence level = 1.96; p = Proportion of patients willing to accept kidney transplant was 66.7% (Takure et al., 2016 e = margin of error = 0.05.

Therefore; the minimum required sample size was 34.

This study comprised 342 ESRD patients on haemodialysis and Stage III-V CKD patients not on haemodialysis.

Participants aged 18 years and over were recruited consecutively from the dialysis units of the Medical department, the National Cardiothoracic centre of the Korle-Bu Teaching Hospital and the central outpatient department. The recruitment took place while patients were waiting for their dialysis sessions.

Patients who had kidney transplant, acute kidney injury and vulnerable participants such as pregnant women and institutionalized individuals were excluded from the study. Participation in this study was completely anonymous and voluntary and written consent was obtained from recruited participants.

### Data collection and tools

Trained research assistants recruited eligible and consented participants for the study. A structured interviewer-administered questionnaire comprising 44 items was completed by each participant. Questionnaire was developed based on information from a previous study [15] and modified to suit the aim of this study. The questionnaire was piloted among 52 patients in a different dialysis treatment centre outside the Korle-Bu Teaching hospital with similar characteristics for face and construct validity. The language and content of the questionnaire were reviewed after pilot testing for easy understanding and administration during data collection.

The final questionnaire was used to obtain basic socio-demographic data (age, gender, educational status, marital status, ethnicity, religion, employment, income, wealth index and living status (for socio-economic status assessment)); social support system (family, friends); health insurance enrollment status; clinical information (comorbidities, duration of dialysis therapy, frequency of dialysis); knowledge, attitude and perception of kidney transplantation. INSPIRIT questionnaire assessed religiosity and spirituality. Participants were assisted to complete the questionnaire in cases where they were unable to complete them on their own. The contingent valuation method (CVM) was used to assess willingness to pay for kidney transplantation.

The CVM is a survey-based, hypothetical and direct method used to assess the monetary valuations of effects of health services [16]. This study adopted the “dichotomous choice format” of contingent valuation. The “dichotomous choice format” assesses the participant’s willingness to pay the existing cost of transplantation ($17550). with only two possible responses (Yes/No) [17,18]. An added follow-up question to the dichotomous approach was posed to all participants who answered “No” to the willingness to pay the existing cost in order to improve on the precision of willingness to pay estimates [19]. This follow up question asked participants if they are willing to pay up to 15% less the existing cost of transplantation ($14918) with response ofYes/No. Participants who answered “No” to the follow up question were then asked to state how much they were willing to pay. All currency conversion in Ghana Cedis was done at December 2018 exchange rate of $ 1 = GHS 5.0712.

The asset-based wealth index gives a composite measure of a participants cumulative living standard. It was calculated by using information collected on participants ownership of the following selected assets, televisions, radio, electricity, refrigerator/freezer/fridge, mobile phone, desktop/laptop computers, fan, motorbike, tractor, bicycles, materials used for housing construction; and types of water access and sanitation facilities, etc. The assets followed the standard used for the Ghana Demographic and Health Surveys [20]. Using principal components analysis, an index was generated to place individuals on a continuous scale of relative wealth. The wealth index was then used to separate all participants into five wealth quintiles– 1–5, with 1 representing poorest and 5 representing wealthiest.

Knowledge level of study participants on kidney transplantation was assessed by asking them to rate their knowledge about kidney transplantation with the following response options: 1 –“I have no knowledge of it”, 2 –“Little”, 3 –“Average”, 4 –“Above average”, 5 –“Well informed”. These ratings were then re-categorized into three leveled: 1 –“Below average” (1 –“I have no knowledge of it”, 2 –“Little”), 2 –“Average”, 3 –“Above average” (4 –“Above average”, 5 –“Well informed”) before being used in the analysis.

### Data analysis

Descriptive statistics on categorical socio-demographic and clinical variables were reported in the form of frequencies and percentages while the continuous variables were presented in means and standard deviation or median with interquartile range where appropriate. Proportion of respondents who were willing to pay any stated amount was reported as a measure of level of willingness to pay. Willingness to pay was dichotomized into 1- willing to pay $17550 for kidney transplantation and 0 –for otherwise. Binary logistic regression model was used to assess the effect of the various independent variables on the willingness pay for kidney transplantation. Penalized maximum likelihood logistic regression and Poisson regression models were also fitted for sensitivity analysis. All statistical tests were set at 5% significance level.

### Ethical consideration

This research was approved by the Korle-Bu Teaching Scientific and Technical Committee (STC) as well as the Institutional Review Board (IRB) with protocol identification number of KBTH-STC 000140/2018. All participant provided written informed consent to take part in the study.

## Results

### Background characteristics of study participants

Three hundred and forty-two (342) study participants were recruited. The average age was 50.2 ± 17.1 years with most (56.7% (194/342)) study participants being male (Table 1). More than half (56.73% (194/342)) of the participants were married whilst almost 40% (138/342) had education up to the tertiary level (Table 1). Forty three percent (147/342) of our study participants were employed and 63% (62.8%, 215/342) were on maintenance haemodialysis (Table 1).

**Table 1:** Background characteristics of study participants at a renal clinic at the Korle-Bu teaching hospital, Accra, Ghana. 2019.

	Frequency	Percent
Age (Mean ± SD)	50.21 ± 17.10	
**Sex**		
Male	194	56.73
Female	148	43.27
**Marital Status**		
Married	194	56.73
Single	81	23.68
Widowed	44	12.87
Divorced	23	6.73
**Educational level**		
No formal education	19	5.56
Primary	109	31.87
Secondary	76	22.22
Tertiary	138	40.35
**Employment Status**		
Unemployed	115	33.63
Employed	147	42.98
Retired	80	23.39
**Wealth quintile**		
1st quintile	69	20.18
2nd quintile	69	20.18
3rd quintile	69	20.18
4th quintile	68	19.88
5th quintile	67	19.59
**Social Supports**		
Number of friends you see or hear from per month: Median (LQ, UQ)	10 (3, 15)	
Number of friends could you call on for help: Median (LQ, UQ)	2 (0, 5)	
Number of family friends could you talk to about personal issues: Median (LQ, UQ)	5 (0, 9)	
Number of family members you can call on for help: Median (LQ, UQ)	3 (2, 5)	
**Religiousity score (Mean ± SD)**	3.84 ± 0.34	
**Physician discussion**		
Yes	99	28.95
No	243	71.05
**CKD stage**		
stage 3	31	9.06
stage 4	63	18.42
stage 5	33	9.65
End stage on dialysis	215	62.87
**Knowledge level**		
Below average	169	49.42
Average	143	41.81
Above average	30	8.77
**Enrolled on health insurance**		
Yes	300	87.72
No	42	12.28
**Type of Insurance (n = 300)**		
NHIS	291	97.00
Private	8	2.67
Both	1	0.33
**Willing to accept transplant**		
No	112	32.75
Yes	230	67.25
**Have other source of income**		
Yes	15	4.39
No	327	95.61

**C:** Responded by only those who had health insurance, UQ: Upper Quartile, uOR: Unadjusted odds ratio, CKD: Chronic Kidney Disease, SD: Standard Deviation.

About 51% (173/342) of study participants rated their knowledge level of kidney transplantation to be at least average. (Table 1). Approximately 67% (230/342) of study participants were willing to accept a kidney transplant if needed.

### Willingness to pay for kidney transplant

**Fig 1:**
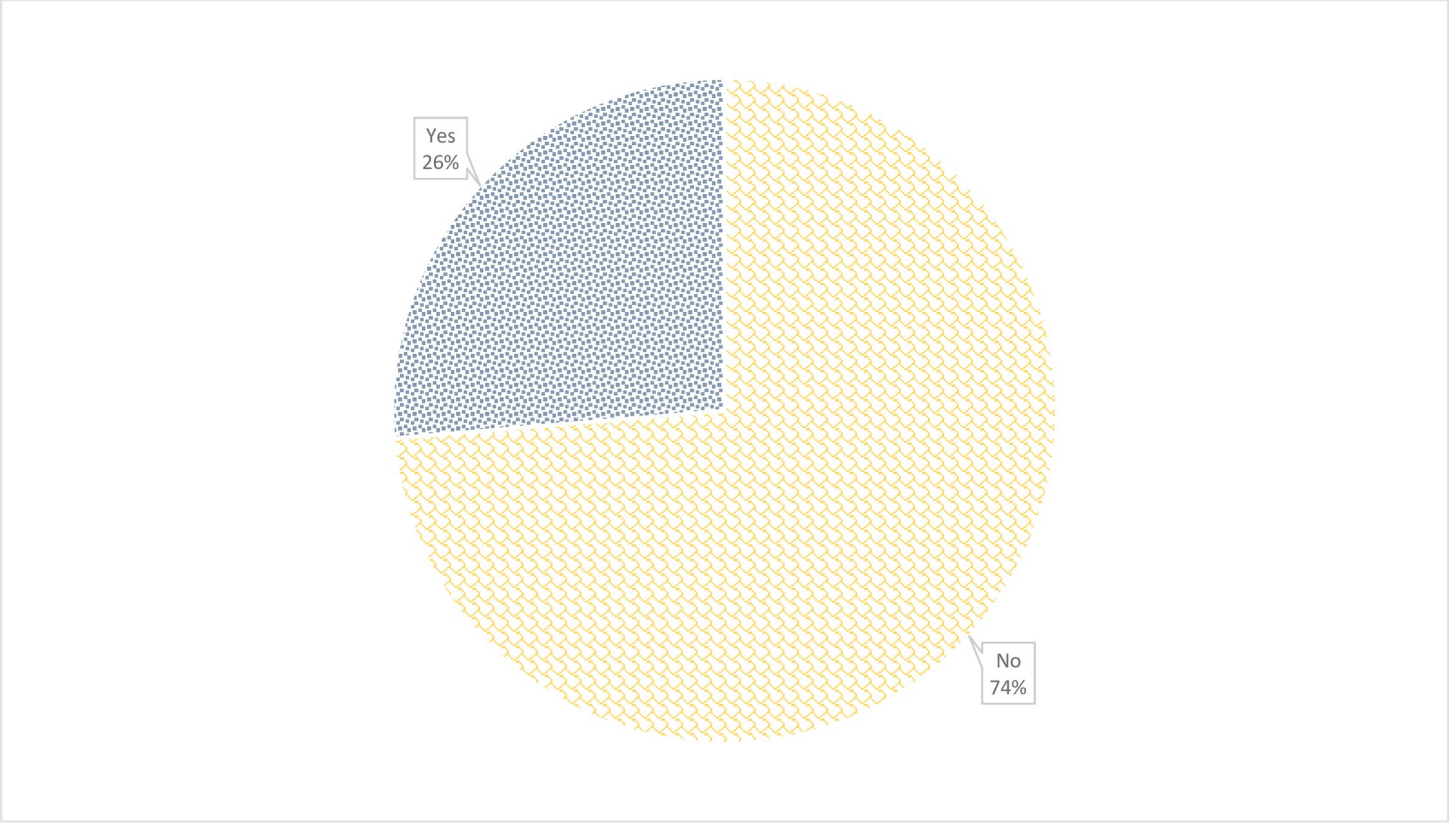
Distribution of participants willingness to pay for kidney transplantation.

Overall, 90 of the 342 study participants (26.3%, 95%CI: 21.7–31.3%) were willing to pay for a kidney transplant at the current going price or more (≥ $ 17,550) (Fig 1). About 66.7% (228/342) willing to pay below $ 17,550 to receive a kidney transplant. However, twenty-three participants (6.7%) were not willing to pay any amount for kidney transplantation as they expect it to be free. Generally, the median amount participants were willing to pay below the going price was $986 (IQR: $197 –$1972). Among those willing to accept (67.3%, 230/342), 29.1% (67/230) were willing to pay for kidney transplant at the prevailing price.

Table 2 shows a Chi-square test which demonstrate a significant association between willingness to pay and the following variables; educational status (p < 0.01), sex (p = 0.014), wealth quintile (p < 0.01), knowledge level about kidney transplantation (p = 0.013), social support with respect family friends one can talk to about personal issues (p = 0.011) and family members one can call on for help (p < 0.01).

**Table 2:** Chi-square test showing association between background characteristics and willingness to pay for kidney transplant.

	Willingness to pay			
	No, n (%)	Yes, n (%)	uOR (95% CI)	P-value
Age(Mean ± SD)	49.67 ± 16.80	51.7 ± 17.95	1.01(0.99–1.02)	0.335
**Sex**				0.014*
Male	133(68.56)	61(31.44)	1	
Female	119(80.41)	29(19.59)	0.53(0.32–0.88)	
**Marital Status**				0.878
Married	141(72.68)	53(27.32)	1	
Single	62(76.54)	19(23.46)	0.82(0.45–1.49)	
Widowed	33(75)	11(25)	0.89(0.42–1.88)	
Divorced	16(69.57)	7(30.43)	1.16(0.45–2.99)	
**Educational level**				< 0.001***
No formal education	18(94.74)	1(5.26)	1	
Primary	91(83.49)	18(16.51)	3.56(0.45–28.39)	
Secondary	64(84.21)	12(15.79)	3.38(0.41–27.73)	
Tertiary	79(57.25)	59(42.75)	13.44(1.74–103.56)	
**Employment Status**				0.159
Unemployed	91(79.13)	24(20.87)	1	
Employed	101(68.71)	46(31.29)	1.73(0.98–3.05)	
Retired	60(75)	20(25)	1.26(0.64–2.49)	
**Wealth quintile**				<0.001***
1st quintile	65(94.2)	4(5.8)	1	
2nd quintile	61(88.41)	8(11.59)	2.13(0.61–7.44)	
3rd quintile	53(76.81)	16(23.19)	4.91(1.55–15.56)	
4th quintile	48(70.59)	20(29.41)	6.77(2.17–21.1)	
5th quintile	25(37.31)	42(62.69)	27.3(8.87–84.05)	
**Social Support**				
Number of friends you see or hear from per month: Median (LQ, UQ)	10(3, 12)	6(2, 15)	1.01(0.99–1.04)	0.391
Number of friends could you call on for help: Median (LQ, UQ)	3(0, 5)	2(0, 5)	1.04(0.99–1.10)	0.142
Number of family friends could you talk to about personal issues: Median (LQ, UQ)	6(0, 10)	1 (0, 8)	0.94 (0.90–0.99)	0.011*
Number of family members you can call on for help: Median (LQ, UQ)	3(2, 5)	4(2, 6)	1.16(1.07–1.26)	0.001**
**Religiousity(Mean ± SD)**	3.85 ± 0.34	3.82 ± 0.36	0.75(0.39–1.46)	
**Physician_discussion**				0.077
Yes	66(66.67)	33(33.33)	1	
No	186(76.54)	57(23.46)	0.6(0.33–1.06)	
**CKD stage**				0.178
stage 3	19(61.29)	12(38.71)	1	
stage 4	52(82.54)	11(17.46)	0.33(0.13–0.89)	
stage 5	24(72.73)	9(27.27)	0.59(0.21–1.7)	
End stage on dialysis	157(73.02)	58(26.98)	0.58(0.27–1.28)	
**Knowledge level**				0.013*
Below average	134(79.29)	35(20.71)	1	
Average	102(71.33)	41(28.67)	1.5(0.82–2.72)	
Above average	16(53.33)	14(46.67)	3.83(1.56–9.4)	
**Willing to accept transplant**				0.092
No	89(79.46)	23(20.54)	1	
Yes	163(70.87)	67(29.13)	1.59(0.93–2.73)	
**Support resources**				0.076
Yes	8(53.33)	7(46.67)	1	
No	244(74.62)	83(25.38)	0.39(0.14–1.10)	

*p < 0.05

**p < 0.01

***p < 0.001

n = frequency; %^a^ represent column percentages; %^b^ represent row percentages; p-values obtained from unadjusted binary logistic regression model, CI: Confidence Interval, SD: Standard Deviation, LQ: Lower Quartile, UQ: Upper Quartile, uOR: Unadjusted odds ratio, CKD: Chronic Kidney Disease.

### Logistic regression model results

From the adjusted logistic model, wealth quintile, social support in terms of Number of family friends one could talk to about personal issues and number of family members one can call on for help were the only factors identified to be significantly predictive of willingness to pay (p-value < 0.05).

Higher wealth quintile was associated with higher odds of being willing to pay for kidney transplant. Thus, the odds of being willing to pay for kidney transplant among participants in the second to fifth quintiles about 1.4, 3.8, 4.2 and 14.1 times higher compared to those in the first quintile respectively (Table 3).

**Table 3:** Logistic regression model showing association between background characteristics and willingness to pay for kidney transplant.

	Logistic regression		Penalized maximum likelihood logistic regression		Poisson regression	
	aOR (95% CI)	P-value	aOR (95% CI)	P-value	aIRR (95% CI)	P-value
Age	1.02(0.98–1.05)	0.327	1.01(0.99–1.04)	0.328	1.01(0.99–1.03)	0.33
**Sex**		0.698		0.726		0.87
Male	1		1		1	
Female	0.87(0.44–1.74)		0.89(0.46–1.71)		0.96(0.58–1.59)	
**Marital Status**		0.344		0.364		0.616
Married	1		1		1	
Single	1.26(0.47–3.34)		1.24(0.49–3.11)		1.17(0.57–2.39)	
Widowed	1.15(0.38–3.5)		1.15(0.41–3.28)		1.08(0.48–2.42)	
Divorced	3.2(0.9–11.38)		2.9(0.89–9.5)		1.73(0.76–3.96)	
**Educational level**		0.07		0.114		0.189
No formal education	1		1		1	
Primary	5.09(0.38–68.92)		3.02(0.35–26.05)		3.06(0.38–24.54)	
Secondary	4.03(0.28–57.35)		2.49(0.27–22.74)		2.57(0.31–21.27)	
Tertiary	10.07(0.72–140.01)		5.59(0.63–49.65)		4.58(0.57–37.01)	
**Employment Status**		0.498		0.519		0.659
Unemployed	1		1		1	
Employed	0.97(0.45–2.07)		0.98(0.48–2.01)		0.95(0.54–1.68)	
Retired	0.54(0.17–1.67)		0.57(0.2–1.66)		0.69(0.3–1.59)	
**Wealth Quintile**		<0.001***		<0.001***		0.002**
1st quintile	1		1		1	
2nd quintile	1.44(0.37–5.56)		1.37(0.39–4.79)		1.58(0.46–5.43)	
3rd quintile	3.84(1.05–13.97)		3.24(0.98–10.73)		3.07(0.97–9.72)	
4th quintile	4.17(1.16–14.98)		3.46(1.06–11.33)		3.35(1.07–10.48)	
5th quintile	14.06(3.9–50.76)		10.24(3.14–33.43)		6.03(1.96–18.58)	
**Social Supports**						
Number of friends you see or hear from per month	1.01(0.96–1.05)	0.809	1(0.97–1.04)	0.82	1(0.97–1.03)	0.951
Number of friends could you call on for help	1.02(0.93–1.12)	0.642	1.02(0.93–1.11)	0.679	1.01(0.96–1.07)	0.633
Number of family friends could you talk to about personal issues	0.93(0.87–0.99)	0.021*	0.94(0.88–0.99)	0.032*	0.96(0.92–1.01)	0.114
Number of family members you can call on for help	1.18(1.06–1.32)	0.002**	1.16(1.05–1.28)	0.004**	1.08(1.02–1.14)	0.014*
**Religiosity**	0.8(0.33–1.94)	0.621	0.84(0.37–1.91)	0.675	0.84(0.46–1.5)	0.548
**Physician discussion**		0.485		0.511		0.556
Yes	1				1	
No	0.77(0.37–1.6)		0.79(0.4–1.58)		0.85(0.5–1.45)	
**CKD stage**		0.376		0.44		0.571
stage 3	1		1		1	
stage 4	0.44(0.13–1.48)		0.48(0.16–1.51)		0.6(0.25–1.44)	
stage 5	0.61(0.16–2.3)		0.63(0.18–2.18)		0.82(0.31–2.17)	
End stage on dialysis	0.94(0.33–2.61)		0.92(0.35–2.43)		0.97(0.47–2.02)	
**Knowledge Level**		0.572		0.609		0.619
Blow average	1		1		1	
Average	1.36(0.66–2.76)		1.33(0.68–2.61)		1.22(0.73–2.05)	
Above average	0.88(0.27–2.9)		0.93(0.31–2.85)		0.93(0.43–2.01)	
**Willing to accept transplant**		0.374		0.432		0.62
No	1		1		1	
Yes	1.35(0.7–2.61)		1.28(0.69–2.39)		1.14(0.69–1.88)	
**Have other source of income**		0.703		0.715		0.837
Yes	1		1		1	
No	0.76(0.19–3.07)		0.79(0.22–2.86)		0.91(0.37–2.23)	

*p < 0.05

**p < 0.01

***p < 0.001

CI: Confidence interval, aOR: Adjusted odds ratio, aIRR: Adjusted incidence-rate ratios, CKD: Chronic Kidney Disease.

With regards to the number of family members you can call on for help, every additional person one participant could call for help was associated with 18% (aOR: 1.18, 95%CI: 1.06–1.32) increase in the odds of being willing to pay for kidney transplant (Table 3).

However, for the number of family friends one could talk to about personal issues, there was a negative relationship between Number of family friends one could talk to about personal issues and the odds of being willing to pay for kidney transplantation. Thus, every additional family friends one could talk to about personal issues was associated with 7% (aOR: 0.93, 95%CI: 0.87–0.99) reduced odds of being willing to pay for kidney transplant (Table 3).

These results from the multiple binary logistic regression model were consistent with those of the adjusted Penalized maximum likelihood logistic regression and Poisson regression models (Table 3).

## Discussion

The present study determined participants’ willingness to pay for kidney transplant and factors that predict their willingness to pay. Overall, nearly a third of participants were willing to pay for kidney transplantation. More than 50% of participants rated their knowledge level on kidney transplantation as average of which a third were willing to pay for kidney transplantation at or above the existing cost. Most of the participants would pay below the current cost with the median willingness to pay at $986 compared to current cost of $17550 and over. Participants in the higher wealth quintile and level of social support in terms of number of family members they could call on for help were more likely to pay for kidney transplant at the existing cost, whilst those who are religious were unlikely to pay for kidney transplant.

To our knowledge, no study examined participants willingness to pay for kidney transplantation among CKD participants or other organ donors. Thus, we reviewed studies that assessed willingness to pay for chronic illness.

Puteh et.al (2017), examined participants’ willingness to pay for medicines for chronic illness in Malaysia and found that 72.2% of respondents were not willing to pay for drug charges and WTP for drugs either for treatment of acute or chronic illness were low with a median of USD 3.8 for drugs per visit [21]. This finding is consistent with the current study where the willingness to pay for kidney transplant service was low for majority of the patients. Another study by Tran et al (2018) in Vietnam showed similar findings of patients’ level of willingness to pay below the expected price of the services [22].

A similar finding was observed in another study by Zhou et al where 77.7% of patients’ WTP for colo-rectal cancer in Guangzhou was < $ 56.00 (below the expected provider price for the service) [23]. In contrast, a study on WTP for depression treatment in primary care centers in USA revealed that the amount patients were willing to pay for treatment of depression was $ 270±187 which represents 9% of the participants’ household income. This was comparable to the cost of other chronic illness and higher than the actual cost of depression treatment [24]. In general, it appears from the above findings that patients are willing to pay charges which are below the expected cost of the services they receive from health service providers.

Although, employment status and educational status did not predict WTP in our study the proxy indicator of wealth (wealth quintile) used to assess socioeconomic status was significantly associated with WTP. The study revealed that higher wealth quintile was associated with higher odds of willingness to pay for kidney transplant. For instance, those in the 5^th^ quintile of wealth status were more than 108 times WTP compared to those in the 1^st^ quintile. This finding is supported in a study by Abate et al which assessed the association between WTP using CVM and socioeconomic status and found a significant association between proxy indicators of wealth, income level and lower medical cost and WTP for Medical care [25]. Again, they found that WTP for heart attack was low in the low-income group and those with low educational status [25]. In another study by Guimarães et al, there was a significant association between WTP for oral insulin delivery system and income level [26]. Many studies on WTP demonstrated that socioeconomic status directly influences WTP for medical care [27–31]. In contrast, WTP for common cold and glaucoma did vary with socioeconomic status in the study by Abate et al [25]. Generally, it appears the high cost of the transplant service and patients’ inability to pay are likely reasons for their unwillingness to pay, as observed. The cost of health care may well be an issue but people who are sick in these low-income areas still need health care. It is well known that kidney transplantation is the ultimate treatment for CKD and it is cost-effective when compared to long-term dialysis [15]. In terms of cost-effectiveness, transplant will be the most ideal treatment option for low-income countries, thus health authorities and non-governmental organizations involved in financing health care must consider financing kidney transplantation especially for this young enterprising workforce of society who are commonly afflicted by CKD [32].

In support of the findings that participants’ willingness to pay for a medical treatment is highly informed by their financial and socio-economic status, Puteh et al showed that higher personal and household income were associated with willingness to pay for drugs [21]. A cross sectional study by Pinto et al looking at the relationship between participant characteristics and satisfaction variables with willingness to pay for inhaled insulin reported that household income and patient satisfaction predicted willingness to pay [33]. Another study reported similar findings where household income predicted patients’ willingness to pay for health care programs [34]. The association between income and willingness to pay is supported by previous studies [23,24].

In this study, over 40% of patients were unemployed, and more than 90% did not have any other regular source of income hence could not readily commit themselves to paying for a service they may not be able to mobilize funds to support. These findings support previous work by Tran et al which reported that willingness to pay among relatively wealthy urban residents was higher compared to rural folks [22]. More so, a multi-center study conducted in developing countries showed a strong correlation between patients financial ability to pay for therapy and their willingness to pay [35]. In line with the findings of the current study, Zhou et al showed that gender and employment status did not predict willingness to pay [23].

Social support in terms of the number of family members participants can call for help significantly predicted their WTP for kidney transplantation. This is an important finding in a setting where patients depend a lot on family members and other donors to pay for health care. Anecdotal evidence in Ghana showed that health care is largely borne by family members such as parents, children, extended family relatives and other donors (employers, churches and philanthropists) as health care is largely financed out-of-pocket [36]. This is compounded by the fact that most of the CKD patients are young between 20 to 50 years of age [32] and mostly lose their jobs once diagnosed with chronic illness. The government can make use of these support systems by providing partial financial support for kidney transplantation to help reduce the financial burden on these generous donors as this may be a step towards establishing sustainable kidney transplant programme in Ghana.

In the current study males were more likely pay for kidney transplant compared to females. However, there was no statistically significant difference between males and females regarding their WTP. This finding is similar to what was observed in a study by Oga et al among diabetic and cardiovascular disease patients where males were 2.3 to 2.5 times more likely to pay a premium for insurance compared to females [37]. This may be the Ghanaian setting where males have more purchasing power and also tend to bear most family expenses.

The study has the following limitations; 1) the cross-sectional nature of the study, did not give us the opportunity verify the association between willingness to pay for kidney transplantation and actual receipt of a transplant as there was no available data on subsequent wait-listing and kidney transplantation to assess; 2) the self-report method used to assess patients’ knowledge on kidney transplantation may introduce errors due to recall bias on possible discussions with physician.

Nevertheless, to our knowledge this study presents a new dimension in that, it is first time contingent valuation was used to assess willingness to pay for kidney transplantation. Furthermore, this is the first study on WTP for a health service including kidney transplantation in Ghana, as result it provides important information on modifiable factors that must be addressed prior to establishment of a sustainable transplant programme in Ghana.

## Conclusion

The level of willingness to pay for kidney transplantation was lower than actual cost among chronic kidney disease patents and only a third of participants were WTP at the current price and above in Korle-Bu Teaching Hospital. The wealth quintile and social support in terms of the number of family members they can call for help were predictive of willingness to pay. These findings may be useful to policy makers and provide an understanding on how to develop pricing models to attain an ideal balance between a cost effective but sustainable kidney transplant programme and improve patient access to this ultimate treatment option in Ghana.

## Supporting information

S1 File (PDF)

S1 Dataset (DTA)

## Abbreviations

CKD: Chronic Kidney Disease
ESRD: End Stage Renal Disease
WTP: Willingness to Pay
CV: Contingent Valuation
