# Supplementary material for: Willingness to pay for kidney transplantation among chronic kidney disease patients in Ghana
Source: PLoS One. 2020 Dec 30;15(12):e0244437. doi: 10.1371/journal.pone.0244437 (PMC7773273; doi:10.1371/journal.pone.0244437)
Supplement: S1 File — (PDF) [file pone.0244437.s001.pdf]

APPENDIX A: STUDY QUESTIONNIARE

Questionnaire ID: ……………….

Date of Survey: ……./……./………
dd mm yyyy

Name of Interviewer: ………………………

DEMOGRAPHICS

1. What is your age?

2. What is your gender?

   1. Male 2. Female

3. What is the highest level of education you have received?

   a. No formal education
   b. Primary/JHS
   c. Senior high School
   d. Tertiary education (University, Vocational etc)
   e. Others….(specify)

4. What is your employment status?

   a. Employed full time
   b. Employed part time
   c. Unemployed
   d. Retired

5. Who earns income to sponsor you? ………………………

6. How many hours each week does the above person work? ………………….

7. What corresponds to the above person(s) current work situation?

   a. Working full time
   b. Working part time
   c. Not working and not looking for work
   d. Unemployed and looking for work
   e. Disabled or retired and not looking for work
   f. Currently in school

8. Do you have other regular sources of income?

   a. Yes
   b. No

9. Since you answered yes to the above question, what sources do you have……………………..

10. Since you answered yes to question 8 specify and estimate the amount ……………………….

11. What is your monthly income from your regular work? ……………………………….

12. How many people are supported by this income? ……………………………………..

13. What is your ethnicity?

   a. Ashanti/Fanti
   b. Ewe
   c. Ga-Adangbe
   d. Dagbani
   e. Hausa
   f. Others-please specify ____

14. What is your current marital status?

   a. Married
   b. Single
   c. Widowed
   d. Divorced
   e. Not married but living with a partner

15. What is your religion

   a. Christian
   b. Muslim
   c. Non-denominational
   d. not religious
   e. others- (specify)____

16. Does your household have?

	Yes	No
Electricity	☐	☐
Radio	☐	☐
Television	☐	☐
Video deck/CD/DVDplayer	☐	☐
Freezer	☐	☐
Mobile Telephone	☐	☐
Non-mobile Telephone	☐	☐
Desktop Computer	☐	☐
Fan	☐	☐
Bicycle	☐	☐
Motorbike	☐	☐
Car	☐	☐
Tractor	☐	☐

Main material of the floor of your house

a. Earth/sand/mud
b. Wood/Palm/Bamboo
c. Cement floor
d. Terrazzo/
e. Ceramic tiles

Main source of drinking water
a. River/stream
b. Water from open well

c. Water from covered well/borehole
d. Piped water
e. Bottled/sachet water

Main method for sewage disposal
a. Dumped around house
b. Refuse damp
c. Burned
d. Collection

Main toilet facility for household members
a. No facility/bush
b. Pitlatrine with slab
c. Ventilated improved pit
d. Latrine Flush Toilet

What type of fuel do your household normally use for cooking?
a. Firewood
b. Saw dust
c. Charcoal
d. Kerosene
e. LP Gas
f. Electricity
g. Other (specify) ………

The following questions concerns your spiritual or religious beliefs and experience

17. How strongly religious (or spiritually-oriented) do you consider yourself?
    a. Not at all
    b. Not very strong
    c. Somewhat strong
    d. Strong

About how often do you spend time on religious or spiritual practices?
a. Once per year
b. Once per month to several times per year
c. Once per week to several times per month
d. Several times per day to several times per week

How often have you felt as though you were very close to a powerful spiritual force?
a. Never
b. Once or twice
c. Several times
d. Often

People have many different images and definitions of the higher power that we often call God. Use your image and your definition of God when answering the following questions

How close do you feel to God?

a. I don't belief in God
b. Not very close
c. Somewhat close
d. Extremely close

Have you ever had an experience that has convinced you that God exists?
a. No
b. I don't know
c. May be
d. Yes

Indicate whether you agree or disagree with the statement “God dwells within you”

a. Definitely disagree
b. Tend to disagree
c. Tend to agree
d. Definitely agree

The next series of questions will focus on questions about your level of social support.

18. How many friends do you see or hear from at least one a month? ……………….

19. How many friends could you call on for help? (by help we mean any form of assistance or support given by a friend such as monetary, emotional, physical or spiritual support) ……………………..

20. How many family friends could you talk to about personal issues? By personal issues we mean any vital or unsettled problem that affects you. This may be related to finance, health, family, work or any aspect of personal life……………………………………………………………………

21. How many family members can you call on for help? ………………………………………..

22. Do you have medical insurance? (NHIS/private medical insurance)**.**

a. Yes
b. No

23. If Yes, what type? (Indicate all that apply)

a. NHIs
b. Private Insurance
c. Both
d. Others (specify)

The next series of questions will focus on knowledge and attitudes towards kidney disease and transplantation.

24. How long have you had kidney disease? ……………………..

25. Do you know what percentage of kidney function you have?

1. >60%
2. 30-60%
3. 15-30%
4. <15%
5. I don’t know

26. Have you heard about kidney transplant before?

  a. Yes
  b. No [Skip to question 33]
  c. Don't Know/Not sure [Skip to question 33]

27. If yes, from who?

  a. My doctor
  b. Friend
  c. Relative
  d. Social media, literature, news
  e. Others (please specify)

28. Has your doctor ever discussed kidney transplant as an alternative to dialysis with you?

  a. Yes
  b. No [Skip to question 33]
  c. Don't Know/Not sure [Skip to question 33]

29. How long did your doctor talk to you about transplant?

  a. Didn't talk about transplant.
  b. <15 minutes
  c. 15-30 minutes
  d. Over 30 minutes.
  e. I don't think the time has come yet.

30. Have you ever been referred for transplant evaluation?

  a. Yes
  b. No
  c. Don't Know/Not sure

31. Are you aware of any transplant centers in centers in Ghana?

  a. Yes
  b. No
  c. Don't Know/Not sure

32. How would you rate your knowledge about kidney transplant?

  a. I have no knowledge of it
  b. Little
  c. Average
  d. Above average
  e. Well informed

33. Do you feel that you need to know more about kidney transplant?

  a. Yes
  b. No
  c. Don't Know/Not sure

34. If there is a class about kidney transplant, would you attend?

a. Yes
b. No
c. I don’t know

35. Do you know the level of kidney function when a transplant can be done?

a. When the kidney function is less than 20ml/min
b. Only after a patient has started dialysis.
c. I don’t know

36. Would you undergo kidney transplant if you are given a chance when the time comes?

a. Yes
b. No
c. Don’t Know/Not sure

37. If you answered “No” to question 36 above, please rank how important these factors are in your decision not to undergo a kidney transplant.

	Not important	Somewhat important	Important	Very important	Don’t know
a. I don't trust the doctors **TRUST DOCTORS**	1	2	3	4	8
b. I need more time to think and learn about it **MORE TIME**	1	2	3	4	8
c. Religious concerns **RELIGIOUS CON**	1	2	3	4	8
d. Complications from transplant **COMPLICATIONS**	1	2	3	4	8
e. Surgical concerns-pain, fear **SURG CONCERNS**	1	2	3	4	8
f. I don’t want somebody else’s organ in my body **SOMEBODY ORGAN**	1	2	3	4	8
g. I don’t think I’ll ever need it. I feel healthy **FEEL HEALTHY**	1	2	3	4	8
h. Financial concerns- not sure how the cost of transplant and medicines will be covered **FINANCIAL CONCERNS**	1	2	3	4	8

i. Other (please specify) ________**OTHER**____

38. Do you think a living person can donate a kidney to patients needing it?

a. Yes
b. No
c. Don’t Know/Not sure

39. Would you prefer a living kidney from a living person or a deceased (a person who has previously died) kidney?

a. Deceased Kidney
b. Living Kidney

c. N/A- I don't want a kidney transplant.
d. No preference

40. If you need a kidney transplant, do you think you would be able to ask someone to donate a kidney to you?

a. Yes
b. No

41. If yes, who would you ask?

a. Close Family (spouse, children, parents, siblings)
b. Relatives
c. Friends
d. All of them Friends or family
e. Others please Specify ______________________

42. If no, why is that?

1........................ I am worried of the effects of losing one kidney in them
2........................ I don't want my relatives to know that I have kidney disease
3........................ I don't have anybody to ask
4........................ I feel uncomfortable asking something for myself
5........................ Not sure how the cost of transplant would be covered
6........................ Others please Specify ____________________
7........................ More than or equal of any two above responses

43. If you had the opportunity, would you have donated your kidneys?

a. Yes[Skip to question 39]
b. No
c. I'm not sure

44. If no, why?

a. Fear of surgery
b. What if my single kidney fail in future
c. I'm not healthy overall.
d. My family won't let me.
e. Others please Specify ______________________

45. Do you think that a person's race can affect their chances of getting a kidney transplant?

a. Yes
b. No [Skip to question 41]
c. I don't know [Skip to question 41]

39.. If you answered yes to question 39 above, what role do you think race plays in getting a kidney transplant?

1. No role
2. I think it will help getting a transplant
3. I think it might delay or limit me from getting a transplant.
4. I don't know/ not sure

40. Quality of life refers to it refers to how the individual's wellbeing may be impacted over time by a disease, a disability, or a disorder. How do you think getting a kidney transplant will affect your Quality of life compared with dialysis?

1. Will not affect the quality of life
2. Improve the quality of life
3. Decrease the quality of life
4. I don't know

Next set of questions assesses your willingness to pay for kidney transplantation

41. Assuming you were to pay 89,000 Ghana cedis per head for a kidney transplant, will you be willing to pay? Yes ☐ No ☐

42. If the premium is set at 75,650 Ghana cedis per head for a kidney transplant, would you be willing to pay? Yes ☐ No ☐

43. If no to questions 41 and 42, then how much are you willing to pay for this treatment? ……………….

44. CKD stage
1. stage 3 or less
2. stage 4
3. stage 5
4. End stage on dialysis

Thank you for taking this survey.
